# Anion-bridged secondary solvation sheaths for stable aqueous zinc batteries

**DOI:** 10.1093/nsr/nwag422

**Published:** 2026-07-10

**Authors:** Xiaosha Cui, Xiao Wang, Ho Seok Park, Zhong-Shuai Wu

**Affiliations:** State Key Laboratory of Catalysis, Dalian Institute of Chemical Physics, Chinese Academy of Sciences, China; State Key Laboratory of Catalysis, Dalian Institute of Chemical Physics, Chinese Academy of Sciences, China; University of Chinese Academy of Sciences, China; School of Chemical Engineering, Sungkyunkwan University (SKKU), Republic of Korea; SKKU Institute of Energy Science & Technology (SIEST), Sungkyunkwan University, Republic of Korea; State Key Laboratory of Catalysis, Dalian Institute of Chemical Physics, Chinese Academy of Sciences, China; University of Chinese Academy of Sciences, China

Aqueous zinc-ion batteries represent a promising frontier in safe and low-cost energy storage [1]. However, their practical application is impeded by the high chemical reactivity of the anode with water [2]. Recent strategies regulate the primary solvation shell by increasing high salt concentrations or organic co-solvents to form a solid electrolyte interphase (SEI) and stabilize the coordination of excess water molecules (H_2_O) [3]. Notably, such a SEI readily forms in high-concentration electrolytes, but it remains elusive in conventional electrolytes owing to strong coordination and the consequent precipitation between Zn^2+^ and anions [4]. Previous interventions often ignore the electron-accepting ability of H_2_O, which could be leveraged to form an anion-bridged secondary solvation structure and disrupt the bond between the Zn^2+^ and anions in the first sheath. It is therefore critical to control the coordination between Zn^2+^ and anions within the secondary solvation sheath, so as to simultaneously achieve high ionic conductivity and a stable SEI.

In a recent study published by *Nature Nanotechnology* titled ‘aqueous electrolyte solutions with anion-bridged secondary solvation sheaths for highly efficient zinc metal batteries,’ Dong and his collaborators [5] proposed a disruptive electrolyte design and a promising universal descriptor, challenging conventional paradigms that emphasize the electron-donating rather than the electron-accepting nature of H_2_O.

**Figure 1.**
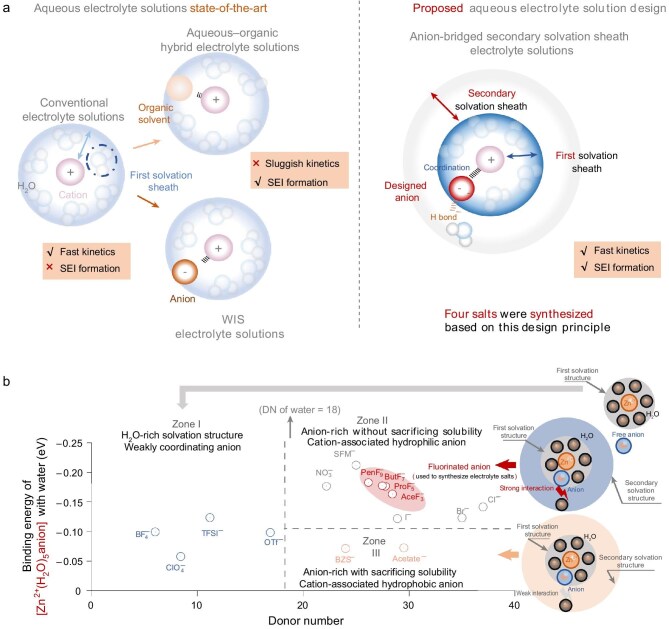
The design principle of anion-bridged secondary solvation sheaths. (a) Schematic representation of the various aqueous electrolyte solutions designs. (b) The anion diagram of DN versus the binding energy between [Zn^2+^(H_2_O)_5_-anion] species and H_2_O in the secondary solvation sheath. Adapt with permission from ref. [5]. Copyright 2026, Springer Nature Limited.

This work proposed the anion donor number (DN) as a quantitative parameter to mediate and lock in long-range solvation interactions (Fig. 1a). Traditionally, H_2_O in the secondary solvation sheath is loosely bound and highly active, serving as the primary trigger for the hydrogen evolution reaction. It was found that anions with a high DN (>18) can penetrate the first layer of the Zn^2+^ solvation shell (Fig. 1b). These coordination anions then act as molecular bridges, using strong hydrogen bonds to anchor H_2_O in the secondary solvation layer on the Zn^2+^ cluster. In this way, water molecules could be effectively immobilized via a multi-scale transformation that spans atomic coordination to nanocluster stabilization. Unlike local high-concentration electrolytes, which rely on inert diluents to preserve solvation structures, and macromolecular additives, which restrict H_2_O mobility through steric effects, this spatial locking mechanism stabilizes H_2_O via a secondary-solvation engineering approach using high-DN anions. This offers a fundamentally different route that simultaneously achieves high ionic conductivity and stable interfacial chemistry.

In addition, this anion-bridged secondary solvation structure governs the interfacial chemistry of the system. As the anions occupy a key position in the first solvation shell, they will be preferentially reduced on the anode surface during the initial cycle, promoting the *in-situ* formation of a ZnF_2_-rich SEI that exhibits zinc-phobic characteristics, a high energy barrier and high Zn^2+^ conductivity. As a result, Zn||Cu batteries achieve a coulombic efficiency of 99.8% after 500 cycles in a 2 M Zn(AceF_3_)_2_ electrolyte. When paired with a NaV_3_O_8_·1.5H_2_O cathode, the assembled full cell delivers a coulombic efficiency of 99.99% and an initial specific energy of ∼130 Wh/kg, demonstrating exceptional cyclability and energy density.

Overall, this work establishes a design principle for anion-bridged secondary solvation shells using fluorinated anions, enabling quantitative control of long-range solvent structures and achieving outstanding electrochemical performance. Defining DN >18 as a quantitative threshold for cluster stability provides a predictive descriptor for electrolyte chemistry across multivalent systems (e.g. Mg, Al, Ca), shifting the field from trial-and-error to data-driven design. This design, which anchors secondary-shell water molecules within nanoclusters, fundamentally redefines the thermodynamic and electrochemical stability window of aqueous batteries. This concept also extends the theoretical framework of aqueous battery chemistry beyond the conventional primary solvation sheath by harnessing the overlooked electron-accepting role of H_2_O, thereby transforming the secondary solvation sheath from a passive spectator into an active structural component that governs interfacial reactions and electrolyte stability. This provides new fundamental insights into the rational design of aqueous electrolytes, effectively decoupling the trade-off between SEI formation and ionic conductivity without resorting to extreme salt concentrations.
